# Is *BRD7* associated with spermatogenesis impairment and male infertility in humans? A case-control study in a Han Chinese population

**DOI:** 10.1186/s12610-021-00139-3

**Published:** 2021-09-02

**Authors:** Tianrong He, Mohan Liu, Dachang Tao, Xiangyou Leng, Zhaokun Wang, Shengyu Xie, Yangwei Zhang, Xinyue Zhang, Xiaolan Tan, Yunqiang Liu, Yuan Yang

**Affiliations:** Department of Medical Genetics, State Key Laboratory of Biotherapy, West China Hospital, Sichuan University, Chengdu, 610041 Sichuan China

**Keywords:** *BRD7*, Rare variant, tagSNP, Spermatogenic failure, Male infertility, BRD7, Variants rares, tagSNP, Altération de la spermatogenèse, Infertilité masculine.

## Abstract

**Background:**

Bromodomain-containing protein 7 (BRD7), a member of the bromodomain-containing protein family, plays important roles in chromatin modification and transcriptional regulation. A recent model of *Brd7*-knockout mice presented azoospermia and male infertility, implying the potential role of *BRD7* in spermatogenic failure in humans. This case-control study aimed to explore the association of the *BRD7* gene with spermatogenic efficiency and the risk of spermatogenic defects in humans.

**Results:**

A total of six heterozygous variants were detected in the coding and splicing regions of the *BRD7* gene in patients with azoospermia. For each of four rare variants predicted to potentially damage *BRD7* function, we further identified these four variants in oligozoospermia and normozoospermia as well. However, no difference in the allele and genotype frequencies of rare variants were observed between cases with spermatogenic failure and controls with normozoospermia; the sperm products of variant carriers were similar to those of noncarriers. Moreover, similar distribution of the alleles, genotypes and haplotypes of seven tag single nucleotide polymorphisms (tagSNPs) was observed between the cases with azoospermia and oligozoospermia and controls with normozoospermia; associations of tagSNP-distinguished *BRD7* alleles with sperm products were not identified.

**Conclusions:**

The lack of an association of *BRD7*-linked rare and common variants with spermatogenic failure implied a limited contribution of the *BRD7* gene to spermatogenic efficiency and susceptibility to male infertility in humans.

**Supplementary Information:**

The online version contains supplementary material available at 10.1186/s12610-021-00139-3.

## Résumé

**Contexte:**

Le bromodomaine contenant la protéine 7 (BRD7), un membre de la famille du bromodomaine contenant des protéines, joue des rôles importants dans la modification de la chromatine et la régulation transcriptionnelle. Un modèle récent de souris *Brd7*-knockout présentait une azoospermie et une infertilité mâle, ce qui implique un rôle potentiel de *BRD7* dans l’altération de la spermatogenèse chez l’homme. Cette étude cas-témoins visait à explorer l’association du gène *BRD7* avec l’efficacité de la spermatogenèse et le risque d’altérations spermatogéniques chez l’homme.

**Résultats:**

Un total de six variants hétérozygotes ont été détectés dans les régions de codage et d’épissage du gène *BRD7* chez les patients présentant une azoospermie. Pour chacun des quatre variants rares prédits pour potentiellement endommager la fonction *BRD7,* nous avons en outre identifié ces quatre variants dans l’oligozoospermie et la normozoospermie. Cependant, nous n’avons observé aucune différence dans les fréquences d’allèle et de génotype des variants rares entre les cas avec altérations de la spermatogenèse et les témoins avec normozoospermie ; les produits du sperme des porteurs de variants étaient semblables à ceux des non-porteurs. Par ailleurs, on a observé une distribution semblable des allèles, des génotypes et des haplotypes de sept polymorphismes simples de nucléotide de balise (tagSNPs) entre les cas avec azoospermie ou oligozoospermie et les témoins normozoospermiques; aucune association n’a pas été identifiée entre les allèles *BRD7* tagSNP-distingués et des produits du sperme.

**Conclusion:**

L’absence d’association des variants rares liés à *BRD7* et des variants communs liés à BRD7 avec les altérations de la spermatogenèse implique une contribution limitée du gène *BRD7* à l’efficacité spermatogénique et à la susceptibilité à l’infertilité masculine chez l’homme.

## Background

Infertility has been a major global public health issue and causes significant psychosocial stress for couples suffering from this condition [1]. It is estimated that approximately 15% of couples suffer from infertility worldwide, and approximately half of infertility cases are caused by male factors [2]. Male infertility due to oligozoospermia (OZ) and azoospermia (AZ) is a common and complex disease. It has been postulated that the cause of infertility in 10–15% of infertile patients with AZ and severe OZ involves genetic factors, and the relevance of genetic anomalies gradually increases with decreasing sperm count [3, 4]. Both chromosomal abnormalities and monogenic mutations could be directly responsible for spermatogenic failure, in which Klinefelter’s syndrome and azoospermia factor (AZF) microdeletion are the most common cytogenetic and molecular genetic causes of spermatogenic failure, respectively [3]. However, the aetiology of approximately 40% of males with spermatogenic failure remains elusive [4], suggesting the significance of further exploring genetic causes of the protein 7 (BRD7), a member of the bromodomain-containing protein family, is highly conserved during evolution and ubiquitously distributed in various tissues with high expression in the testes of humans [5]. A recent study reported *Brd7* knockout, causing AZ, and complete arrest of spermatogenesis at step 13 in mice [6]. Compared with BRD7^+/+^ mice, BRD7^−/−^ mice showed a decrease in testicular size and seminiferous tubule diameter [6]. Furthermore, BRD7^−/−^ mice had morphologically abnormal round spermatids, elongating spermatids and denatured condensed spermatids with irregular head shapes and deformed acrosomes [6]. Remarkably, BRD7 expression in the testis was reduced significantly in patients with idiopathic AZ relative to men with normozoospermia (NZ) [6]. These findings suggest a vital role of the *BRD7* gene in spermatogenesis. In this case, it would be interesting to determine whether the *BRD7* gene is associated with the risk of spermatogenic failure and male infertility in humans. In the present study, we detected rare and common variants of *BRD7* in 315 infertile patients with spermatogenic failure and 995 men with NZ. Our results implied a limited contribution of the *BRD7* gene to susceptibility to spermatogenic failure and male infertility in humans.

## Materials and methods

### Participants

The sample size for the case-control study was calculated using QUANTO1.2 software (Jim Gauderman and John Morrison, USA). The parameters of the type I error rate and statistical power were set at 0.05 and 0.80, respectively. The evaluated sample size was at least 314 for the case group when the size ratio of the patients and controls was 1:3. According to the sample size, we recruited 315 unrelated infertile men with idiopathic spermatogenesis impairment and 995 normozoospermic men (couple infertility due to female factors) from two affiliated hospitals of Sichuan University and Chengdu Women’s and Children’s Central Hospital between 2015 and 2020.

The diagnosis of all patients was based on standard clinical procedures, including history and physical examination, semen analysis, serum hormone analysis, ultrasound evaluation and genetic testing [7]. All of the participants underwent at least two semen analyses. Based on World Health Organization guidelines [8], AZ is defined when no sperm is found under the microscope after the semen is centrifuged (3000×g) for 15 min. OZ is defined as sperm concentration (SC) < 15 × 10^6^/ml and total sperm count (TSC) < 39 × 10^6^/ejaculate. NZ is defined as SC > 15 × 10^6^/ml, TSC > 39 × 10^6^/ejaculate and normal sperm motility and morphology. Serum hormones, including follicle-stimulating hormone (FSH), luteinizing hormone (LH) and testosterone (T), were detected in individuals. Patients with alcohol or drug abuse, karyotype abnormalities, AZF microdeletions, hypogonadotrophic hypogonadism, cryptorchidism, varicocele, seminal ductal obstruction, testicular trauma and tumours were excluded based on evaluation with standard clinical procedures. The case group included 142 patients with nonobstructive azoospermia (NOA) and 173 patients with OZ aged 26 to 46 years. The patients with NOA included 51 with hypospermatogenesis (8 ~ 9 scores), 30 with spermatid arrest (6 ~ 7 scores), 22 with spermatocyte arrest (4 ~ 5 scores), 16 with spermatogonia arrest (3 scores), and 23 with Sertoli cell-only syndrome (2 scores) according to the Johnsen score and predominant histopathologic pattern [9]. The controls with NZ ranged in age from 22 to 45 years old. The semen and hormonal parameters of the subjects are shown in Table 1. This study was approved by the Biomedical Research Ethics Committee of West China Hospital, Sichuan University (No. 783), and written informed consent was obtained from each participant.

**Table 1:** The basic characteristics of the study subjects

Parameters	Patients		Controls
	OZ (***n*** = 173)	AZ (***n*** = 142)	NZ (***n*** = 995)
BMI (kg/m^2^)^**a**^	22.89 ± 2.25	22.76 ± 1.96	22.34 ± 2.67
SC (n × 10^6^/ml)^**b**^	6.60 (2.55–10.86)	/	61.46 (40.95–91.88)
TSC (n × 10^6^/ejaculate)^**b**^	15.88 (5.87–27.13)	/	212.07 (133.38–317.08)
Total molitility (PR + NP, %) ^**b**^	38.24 (28.00–53.39)	/	64.56 (50.77–76.70)
FSH (mIU/ml) ^**a**^	5.73 ± 3.69	8.77 ± 3.64	4.83 ± 1.73
LH (mIU/ml) ^**a**^	5.72 ± 3.47	6.93 ± 2.88	4.87 ± 2.01
T (ng/ml) ^**a**^	4.81 ± 1.59	4.52 ± 2.05	5.12 ± 1.63

*OZ* oligozoospermia, *AZ* azoospermia, *NZ* normozoospermia, *BMI* Body mass index, *SC* Sperm concentration, *TSC* Total sperm count, *PR* progressive motility (grades a + b), *NP* non-progressive motility (grade c), *FSH* Follicle-stimulating hormone, *LH* Luteinizing hormone, T Testosterone. ^a^ Mean ± standard deviation, ^b^ Median and interquartile range

### Detection of rare variants in the coding region and splice site of *BRD7*

Genomic DNA was collected from whole blood using a Blood DNA Purification Kit (BioTeke, China). The quality and concentration of DNA samples were assessed by 1% agarose gel electrophoresis. For the 142 patients with NOA, all seventeen exons of *BRD7* (NG_023418) including splice sites were amplified by polymerase chain reaction (PCR), and the PCR primer information is shown in Supplementary table 1. Sanger sequencing of the PCR product was performed on a 3700XL System (Applied Biosystems, USA).

Detected variants with a minor allele frequency (MAF) < 1% in the Genome Aggregation Database (gnomAD) [10] and 1000 Genomes Project [11] were classified as ‘rare variants’. Among these variants, the influence of a missense variant on gene function was predicted by three in silico algorithms, including SIFT [12], PolyPhen-2 [13] and Mutation Taster [14], and the influence of synonymous variants and a variant in splice site on RNA splicing was predicted by two in silico algorithms, including MaxEntScan [15] and Human Splicing Finder [16]. For the rare variants predicted to potentially damage the function of *BRD7* by at least two of three algorithms (SIFT, PolyPhen-2 and Mutation Taster) or one of two algorithms (MaxEntScan and Human Splicing Finder), further genotyping was conducted in 173 infertile males with OZ and 995 controls with NZ by Sanger sequencing.

### Genotyping of the common variants in *BRD7*

The genotypes of single nucleotide polymorphisms (SNPs) within 10 kb are usually associated with the same or similar effects [17, 18], and a single tagSNP could represent the information of more SNPs in the region. Currently, tagSNP selection is mostly based on linkage disequilibrium (LD) [19]. LD, a nonrandom association of alleles at a pair of loci, is quantified by the value of D′ or r^2^ [17, 20]. The value of r^2^ is directly related to the statistical power of detecting unassayed loci and disease-associated polymorphisms [17]. When the value of r^2^ ≥ 0.8, two loci are regarded as exhibiting a strong LD [19]. In the present study, we extracted *BRD7* genotype data from 2 kb upstream of the transcription start site to 2 kb downstream of the transcription stop site from the 1000 Genomes Project database. The tagSNPs were screened and evaluated using Haploview 4.2 software (Broad Institute of MIT and Harvard, USA). Based on the data of Han Chinese individuals in Beijing, a total of seven tagSNPs, rs7196135, rs117164075, rs76946718, rs1062348, rs79483509, rs62029995 and rs11644238, were eventually selected with MAF > 5% and LD value of r^2^ ≥ 0. 8. In theory, these tagSNPs could capture greater than 90% of the targeted *BRD7* alleles at an r^2^ threshold of 0.8.

Genotyping of the tagSNPs was performed for 315 infertile patients with impaired spermatogenesis and 995 controls with NZ using a SNPscan™ Kit (Genesky Biotechnologies, China). As described previously [21], the genotypes of the tagSNPs were identified by double-ligation and multiplex fluorescence PCR, and the results were analysed using GeneMapper 4.1 software (Applied Biosystems, USA). For quality control, 10% of the total samples were randomly selected for the second test with a concordance rate of 100%. Moreover, 5% of the samples were confirmed to have tagSNP genotypes by Sanger sequencing, producing 100% identity.

### Statistical analysis

The distribution of semen parameters, including SC, TSC and motility, was analysed using the Kolmogorov-Smirnov test or descriptive statistical index in SPSS 17.0 software (SPSS Inc., USA). The Hardy-Weinberg equilibrium (HWE) test was performed for each tagSNP using PLINK 1.9 software (Shaun Purcell, USA). The genotype distributions and allele frequencies of the rare variants and tagSNPs were compared between patients and controls using Pearson’s χ2 test or Fisher’s exact test in SPSS 17.0 software. LD analysis of the tagSNPs was conducted using Haploview 4.2 software. Haplotype analysis of the tagSNPs was performed using SHEsis software [22]. The Mann-Whitney U or Kruskal-Wallis test was performed to compare the distribution differences of SC and TSC among different genotypes of patients with OZ and fertile men. Continuous variables are presented as the mean ± standard deviation of the mean (mean ± SD) or median and interquartile range, and categorical variables are presented as frequencies (%). For all statistical tests, *P* < 0.05 was considered to be statistically significant. In addition, the Bonferroni method was applied to adjust for multiple testing by dividing the critical level of significance by the number of comparisons.

## Results

First, we detected variants in the coding region and splice site of *BRD7* in 142 patients with NOA. As a result, a total of six exonic and splicing variants were classified as heterozygous (Table 2). The properties of these variants were evaluated with publicly available population databases and in silico tools. After excluding one synonymous variant (rs201820448) without supporting evidence for its influence on RNA splicing and another (rs1062348) with MAF > 1% in the East Asian population of 1000 Genomes and gnomAD databases, the remaining four rare variants (Supplementary Fig. 1), including rs116422109, rs202057136, rs115302634 and rs188183810, were further subjected to genotyping by Sanger sequencing in 173 infertile males with OZ and 995 normozoospermic men. The four rare variants were also found in the heterozygous state in 173 patients with OZ and 995 controls. The genotype distributions of these variants were in accordance with HWE in both the patient and control groups (Supplementary Table 2). Our results showed a similar distribution of alleles and genotypes of these variants between 995 controls and 315 infertile patients (142 with NOA and 173 with OZ) (Table 3). The human *BRD7* gene is mainly expressed in the nuclei of primary spermatocytes and round spermatids [6], implying that the impaired function of BRD7 may cause spermatocyte or spermatid arrest. Thus, we further compared the distribution of alleles and genotypes between the controls with NZ and NOA patients with either of the two pathological phenotypes in the testis. However, we failed to identify any significant difference in the distributions of alleles and genotypes of these variants between the two groups (Table 3). Further comparison did not reveal any difference in the sperm products between carriers of the variants and noncarriers (Table 4).

**Table 2:** The bioinformatics analysis of exonic and splicing variants detected in 142 patients with AZ

Rs	Variant^**a**^	Consequence	In silico predictive algorithm					MAF in the databases	
			SIFT	PolyPhen-2	Mutation Taster	MaxEntScan	Human Splicing Finder	1000 Genomes_EAS	gnomAD_EAS
rs1062348	c.846C > T	Synonymous	/	/	/	/	/	0.497	0.499
rs116422109	c.537 T > C	Synonymous	/	/	/	NSI	SSC	0.005	0.005
rs202057136	c.592-9A > G	Splicing	/	/	/	SSC (−0.12)	NSI	0.001	0.002
rs115302634	c.1796A > G	Missense	Tolerated (0.127)	Probably damaging (0.994)	Disease causing(0.99)	/	/	0.003	0.004
rs201820448	c.1458 T > C	Synonymous	/	/	/	NSI	NSI	0.004	0.004
rs188183810	c.1077C > T	Synonymous	/	/	/	SSC (−0.04)	SSC	0.005	NA

The exons and their splice sites of bromodomain containing 7(*BRD7*) were amplified by PCR and products were detected using Sanger sequencing

*AZ* azoospermia, *MAF* Minor allele frequency, *gnomAD* Genome Aggregation Database, *EAS* East Asia, *NSI* No significant impact, *SSC* Splice site changes. ^a^The variants were identified in the heterozygous state in patients with AZ

**Table 3:** Comparison of allele and genotype frequencies of the rare variants between patients with OZ or AZ and controls with NZ

Rs	Allele/Genotype	Patients					Controls	***P***-values				
		Total (***n*** = 315)	OZ (***n*** = 173)	AZ1 + AZ2 (***n*** = 142)	AZ1 (***n*** = 52)	AZ2 (***n*** = 90)	NZ (***n*** = 995)	[1]	[2]	[3]	[4]	[5]
rs116422109	T	625 (99.2)	343 (99.1)	282 (99.3)	102 (98.1)	180 (100)	1977 (99.3)	0.782	0.720	1.000	0.169	0.617
	C^**a**^	5 (0.8)	3 (0.9)	2 (0.7)	2 (1.9)	0 (0.0)	13 (0.7)					
	TT	310 (98.4)	170 (98.3)	140 (98.6)	50 (96.2)	90 (100)	982 (98.7)	0.781	0.719	1.000	0.168	0.616
	TC	5 (1.6)	3 (1.7)	2 (1.4)	2 (3.8)	0 (0.0)	13 (1.3)					
rs202057136	A	627 (99.5)	344 (99.4)	283 (99.6)	104 (100)	179 (99.4)	1981 (99.5)	1.000	0.672	1.000	1.000	0.580
	G^**a**^	3 (0.5)	2 (0.6)	1 (0.4)	0 (0.0)	1 (0.6)	9(0.5)					
	AA	312 (99.0)	171 (98.8)	141 (99.3)	52 (100)	89 (98.9)	986 (99.1)	1.000	0.671	1.000	1.000	0.581
	AG	3 (1.0)	2 (1.2)	1 (0.7)	0 (0.0)	1 (1.1)	9 (0.9)					
rs115302634	A	627 (99.5)	345 (99.7)	282 (99.3)	103 (99.0)	179 (99.4)	1982 (99.6)	0.732	1.000	0.361	0.368	0.542
	G^**a**^	3 (0.5)	1 (0.3)	2 (0.7)	1 (1.0)	1 (0.6)	8 (0.4)					
	AA	312 (99.0)	172 (99.4)	140 (98.6)	51 (98.1)	89 (98.9)	987 (99.2)	0.732	1.000	0.361	0.369	0.543
	AG	3 (1.0)	1 (0.6)	2 (1.4)	1 (1.9)	1 (1.1)	8 (0.8)					
rs188183810	C	628 (99.7)	345 (99.7)	283 (99.6)	104 (100)	179 (99.4)	1988 (99.9)	0.246	0.382	0.330	1.000	0.229
	T^**a**^	2 (0.3)	1 (0.3)	1 (0.4)	0 (0.0)	1 (0.6)	2 (0.1)					
	CC	313 (99.4)	172 (99.4)	141 (99.3)	52 (100)	89 (98.9)	993 (99.8)	0.246	0.382	0.330	1.000	0.229
	CT	2 (0.6)	1 (0.6)	1 (0.7)	0 (0.0)	1 (1.1)	2 (0.2)					

*P*-values were calculated using Fisher’s exact test

*OZ* oligozoospermia, *AZ1* azoospermia with spermatid or spermatocyte arrest, *AZ2* azoospermia with hypospermatogenesis, spermatogonia arrest or Sertoli cell-only syndrome, *NZ* normozoospermia. Controls vs. [1] Total patients, [2] OZ, [3] AZ1 + AZ2, [4] AZ1, [5] AZ2. ^a^The variants were identified in the heterozygous state in the subjects

**Table 4:** Comparison of sperm products among different genotypes of the rare variants

Rs	Genotype	No. subjects	Median of SC (25th–75th percentile) (n × 10^**6**^/ml)	***P***-values	Median of TSC (25th–75th percentiles) (n × 10^**6**^/ejaculate)	***P***-values
rs116422109	TT	1152	49.14(18.53–79.62)	0.899	159.40(52.12–274.13)	0.902
	TC	16	38.63(7.74–81.67)		155.42(20.28–311.51)	
rs202057136	AA	1157	49.09(18.36–79.96)	0.829	159.40(51.94–274.89)	0.908
	AG	11	47.27(25.32–71.43)		132.04(56.75–227.02)	
rs115302634	AA	1159	49.14(18.40–79.68)	0.445	159.57(51.96–275.03)	0.532
	AG	9	44.46(21.31–81.98)		129.40(47.00–200.77)	
rs188183810	CC	1165	49.19(18.55–79.89)	0.273	166.14(64.62–279.00)	0.745
	CT	3	27.28(7.58–56.64)		71.67(5.02–271.81)	

*P*-values were calculated using Mann-Whitney U test. *SC* Sperm concentration, *TSC* Total sperm count

To further explore the association of the common variants of *BRD7* with spermatogenesis failure, we identified seven *BRD7*-linked tagSNPs and performed genotyping in 315 patients with NOA or OZ and 995 controls with NZ. The genotype distributions of the seven common SNPs were in accordance with HWE in both the patient and control groups (Supplementary table 3), suggesting that the study sample is representative of the population. As shown in Table 5, the distribution of alleles and genotypes of the seven tagSNPs was similar between patients with NOA or OZ and fertile male controls.

**Table 5:** Comparison of allele and genotype frequencies of the tagSNPs between patients with OZ or AZ and controls with NZ

TagSNPs	Allele/Genotype	Patients			Controls	***P***-values		
		Total (n = 315)	OZ (n = 173)	AZ (n = 142)	NZ (n = 995)	[1]	[2]	[3]
rs7196135	G	176 (27.9)	98 (28.3)	78 (27.5)	578 (29.0)	0.592 ^a^	0.785 ^a^	0.582 ^a^
	A	454 (72.1)	248 (71.7)	206 (72.5)	1412 (71.0)			
	GG	28 (8.9)	15 (8.7)	13 (9.2)	79 (8.0)	0.423 ^a^	0.766 ^a^	0.442 ^a^
	GA	120 (38.2)	68 (39.3)	52 (36.6)	420 (42.2)			
	AA	167 (52.9)	90 (52.0)	77 (54.2)	496 (49.8)			
rs117164075	T	75 (11.9)	40 (11.6)	35 (12.3)	268 (13.5)	0.311 ^a^	0.333 ^a^	0.596 ^a^
	C	555 (88.1)	306 (88.4)	249 (87.7)	1722 (86.5)			
	TT	5 (1.6)	2 (1.2)	3 (2.1)	15 (1.5)	0.493 ^b^	0.655 ^b^	0.515 ^b^
	TC	65 (20.6)	36 (20.8)	29 (20.4)	238 (23.9)			
	CC	245 (77.8)	135 (78.0)	110 (77.5)	742 (74.6)			
rs76946718	T	54 (8.6)	32 (9.2)	22 (7.7)	180 (9.0)	0.716 ^a^	0.903 ^a^	0.472 ^a^
	C	576 (91.4)	314 (90.8)	262 (92.3)	1810 (91.0)			
	TT	1 (0.3)	0 (0.0)	1 (0.7)	7 (0.7)	0.895 ^b^	0.619 ^b^	0.664 ^b^
	TC	52 (16.5)	32 (18.5)	20 (14.1)	166 (16.7)			
	CC	262 (83.2)	141 (81.5)	121 (85.2)	822 (82.6)			
rs1062348	A	306 (48.6)	172 (49.7)	134 (47.2)	993 (49.9)	0.561 ^a^	0.948 ^a^	0.392 ^a^
	G	324 (51.4)	174 (50.3)	150 (52.8)	997 (50.1)			
	AA	72 (22.9)	40 (23.1)	32 (22.5)	243 (24.4)	0.832 ^a^	0.862 ^a^	0.646 ^a^
	AG	162 (51.4)	92 (53.2)	70 (49.3)	507 (51.0)			
	GG	81 (25.7)	41 (23.7)	40 (28.2)	245 (24.6)			
rs79483509	C	134 (21.3)	71 (20.5)	63 (22.2)	446 (22.4)	0.547 ^a^	0.434 ^a^	0.931 ^a^
	T	496 (78.7)	275 (79.5)	221 (77.8)	1544 (77.6)			
	CC	16 (5.1)	5 (2.9)	11 (7.7)	47 (4.7)	0.619 ^a^	0.547 ^a^	0.136 ^a^
	CT	102 (32.4)	61 (35.3)	41 (28.9)	352 (35.4)			
	TT	197 (62.5)	107 (61.8)	90 (63.4)	596 (59.9)			
rs62029995	G	130 (20.6)	65 (18.8)	65 (22.9)	383 (19.2)	0.444 ^a^	0.841 ^a^	0.149 ^a^
	C	500 (79.4)	281 (81.2)	219 (77.1)	1607 (80.8)			
	GG	15 (4.8)	6 (3.5)	9 (6.3)	40 (4.0)	0.742 ^a^	0.942 ^a^	0.316 ^a^
	GC	100 (31.7)	53 (30.6)	47 (33.1)	303 (30.5)			
	CC	200 (63.5)	114 (65.9)	86 (60.6)	652 (65.5)			
rs11644238	C	140 (22.2)	76 (22.0)	64 (22.5)	424 (21.3)	0.626 ^a^	0.783 ^a^	0.637 ^a^
	A	490 (77.8)	270 (78.0)	220 (77.5)	1566 (78.7)			
	CC	15 (4.8)	8 (4.6)	7 (4.9)	45 (4.5)	0.879 ^a^	0.995 ^a^	0.892 ^a^
	CA	110 (34.9)	60 (34.7)	50 (35.2)	334 (33.6)			
	AA	190 (60.3)	105 (60.7)	85 (59.9)	616 (61.9)			

*P*-values were calculated using Chi-squared test^a^ or Fisher’s exact test^b^. *OZ* oligozoospermia, *AZ* azoospermia, *NZ* normozoospermia, *SNP* Single nucleotide polymorphism. Controls vs. [1] Total patients, [2] OZ, [3] AZ

**Fig. 1:**
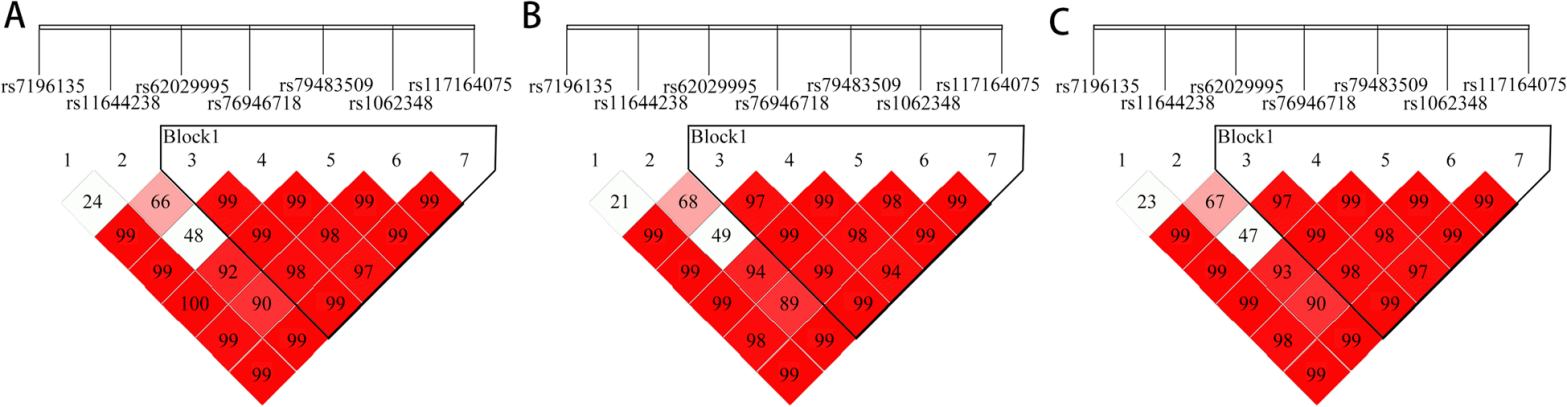
The linkage disequilibrium (LD) pattern of seven *BRD7*-linked tagSNPs in the azoospermia, oligozoospermia and control groups. Legend: Five of the seven tagSNPs, rs62029995, rs76946718, rs79483509, rs1062348 and rs117164075, formed a haploid block with strong linkage disequilibrium in both groups of patients and the controls. LD was evaluated with D’ values. **a** Control group with normozoospermia; **b** Oligozoospermia group; **c** Azoospermia group

Typically, a haplotype composed of SNPs may lead to a larger joint effect on complex traits compared with that noted for single-marker analysis [23]. Therefore, we next conducted pairwise LD analysis of the tagSNPs using Haploview 4.2 software. The results showed that five of the seven tagSNPs, rs62029995, rs76946718, rs79483509, rs1062348 and rs117164075, formed a haploid block that exhibited a strong LD in both patients and controls (Fig. 1). Haplotype analysis with SHEsis software predicted six haplotypes of the haploid block with a frequency of greater than 0.03. However, we did not identify any significant difference in the distribution of these haplotypes between patients with spermatogenesis failure and controls with NZ (Table 6).

**Table 6:** Comparison of the haplotype frequencies between patients with OZ or AZ and controls with NZ

Haplotypes	Patients			Controls	***P***-values		
	Total (***n*** = 315)	OZ (***n*** = 173)	AZ (***n*** = 142)	NZ (***n*** = 995)	[1]	[2]	[3]
CCCGC	0.096	0.098	0.092	0.092	0.824	0.763	0.990
CCCGT	0.123	0.121	0.126	0.134	0.506	0.519	0.740
CCTAC	0.496	0.507	0.483	0.506	0.630	0.939	0.490
CCTGC	0.076	0.088	0.059	0.07	0.701	0.280	0.513
GCTGC	0.121	0.098	0.151	0.108	0.422	0.564	0.051
GTTGC	0.086	0.088	0.084	0.084	0.860	0.814	0.991

The haplotypes were reconstructed by Haploview software. *P*-values were calculated using Chi-squared test. *OZ* oligozoospermia, *AZ* azoospermia, *NZ* normozoospermia. Controls vs. [1] Total patients, [2] OZ, [3] AZ

Furthermore, we investigated the correlation between *BRD7* and sperm products, including SC and TSC. The results showed that men with any *BRD7* alleles distinguished by the seven tagSNPs presented similar SC and TSC (Table 7), further implying the absence of the association of *BRD7* tagSNPs with susceptibility to spermatogenic failure.

**Table 7:** Comparison of sperm products among different genotypes of tagSNPs

TagSNPs	Genotype	No. subjects	Median of SC (25th -75th percentiles) (n × 10^**6**^/ml)	***P***-values	Median of TSC (25th -75th percentiles) (n × 10^**6**^/ejaculate)	***P***-values
rs7196135	GG	94	53.35 (11.60–80.74)	0.473	154.77 (28.35–266.32)	0.556
	GA	488	50.96 (19.08–80.80)		167.40 (68.98–279.29)	
	AA	586	46.95 (16.21–79.32)		156.75 (47.08–274.28)	
rs117164075	TT	17	48.07 (27.36–82.11)	0.146	134.81 (25.88–237.52)	0.183
	TC	274	53.85 (23.49–84.83)		170.12 (70.85–289.61)	
	CC	877	47.38 (16.05–78.88)		159.93 (47.76–271.37)	
rs76946718	TT	7	50.67 (23.97–70.07)	0.626	105.16 (26.14–171.25)	0.523
	TC	198	50.58 (19.10–85.73)		178.90 (54.92–300.45)	
	CC	963	48,61 (17.86–79.03)		158.53 (51.21–271.26)	
rs1062348	AA	283	48.73 (21.87–79.68)	0.756	164.74 (63.86–282.19)	0.514
	AG	599	47.66 (16.23–79.96)		158.43 (53.49–274.30)	
	GG	286	49.63 (18.42–78.68)		159.23 (48.00–273.28)	
rs79483509	CC	52	49.59 (20.45–82.81)	0.220	142.04 (31.08–269.80)	0.532
	CT	413	53.44 (20.92–81.19)		166.63 (68.98–276.66)	
	TT	703	46.95 (16.23–79.11)		156.20 (47.74–274.49)	
rs62029995	GG	46	47.16 (16.95–84.6)	0.406	149.14 (31.68–275.15)	0.237
	GC	356	45.53 (18.36–78.54)		156.42 (50.27–270.95)	
	CC	766	51.33 (22.09–80.64)		165.41 (71.55–279.62)	
rs11644238	CC	53	44.72 (15.85–83.19)	0.331	146.31 (45.10–283)	0.288
	CA	394	47.18 (16.03–77.81)		159.94 (48.45–267.76)	
	AA	721	50.56 (20.42–81.64)		164.99 (64.26–279.45)	

*P*-values were calculated using Kruskal-Wallis test. *SNP* Single nucleotide polymorphism, *SC* Sperm concentration, *TSC* Total sperm count

## Discussion

Spermatogenesis is a complex process involving approximately 2000 genes [4, 24]. By studying human patients with spermatogenic failure, some autosome-linked gene variants have been demonstrated to cause central hypogonadism, monomorphic teratozoospermia or asthenospermia [4]. In recent years, the reproductive investigation of gene-knockout mice has suggested more candidate genes for spermatogenic failure [25], providing an additional clue for the aetiological study of spermatogenic failure in humans. In this case, it is encouraged to clarify the contribution of these genes to spermatogenic failure and male infertility in humans when considering the similarity of function between mouse and human genes [26].

*BRD7* plays various roles in cellular biological processes, such as transcriptional regulation, chromatin modification and cell cycle control [27–29]. As a catalytic subunit of the switch/sucrose nonfermenting (SWI/SNF) complex, brahma-related gene 1 (BRG1) facilitates DNA double-strand break repair and recombination during meiosis in the male germline [30], and *BRD7* is a subunit of polybromo-associated BRG1-associated factor-specific SWI/SNF and is essential for the activation and repression of target genes in embryonic stem cells [31]. In addition, as a protein recognition module, the bromodomain can bind acetyllysine residues on the histone tail, which is a pivotal mark of epigenetic regulation [32, 33]. Remarkably, BRD7 is highly expressed in the pachytene stage to the round spermatid stage during mouse spermatogenesis, which is similar to that in humans, and *Brd7*^*−/−*^ male mice present AZ and male infertility [6]. Interestingly, a study reported that whole-body BRD7 knockout in mice caused embryonic lethality at mid-gestation, suggesting a pivotal role for BRD7 during growth and development [34]. This discrepancy between the two studies was probably due to the different knockout systems. The former BRD7-knockout mice were obtained using the Cre/loxP and flp/FRT recombination systems, which both conditionally and globally destroyed *BRD7* [6]. In the Cre/loxP system, Cre expression is controlled by the EII α promoter, and minor leakage of the Cre/EII α promoter may lead to low *BRD7* expression that is sufficient to allow knockout mice to survive and cause male infertility [35, 36]. Moreover, another bromodomain protein, bromodomain testis-specific protein (BRDT), has been reported to be involved in susceptibility to spermatogenesis impairment in humans [37]. These findings imply that *BRD7* may be a potential candidate gene for human spermatogenesis impairment.

To explore the association of *BRD7* with human spermatogenic failure, we comprehensively investigated the influence of rare and common variants of *BRD7* on the spermatogenic phenotype in 315 infertile patients with AZ or OZ and 995 males with NZ in the present study. However, we did not identify any rare variants of *BRD7* that could impair sperm production to influence the risk of spermatogenic failure in our population. Regarding the common variants of *BRD7*, we failed to obtain any evidence for the association of their alleles with spermatogenic efficiency and susceptibility to spermatogenic failure. Collectively, our findings imply a limited contribution of *BRD7* to human male infertility. This observation may be reasonable when considering that *BRD7* may have partial functional redundancy with other genes during spermatogenesis in humans; thus, it is potentially nonessential for spermatogenesis in humans [38, 39].

Several limitations of the present study should be noted: (i) Approximately 2000 genes play a role in spermatogenesis, and it is highly possible that only a small number of patients with AZ are likely to carry two pathogenic alleles of *BRD7*. These patients may not be detected in the limited number of AZ samples. Thus, the spermatogenic phenotype of complete loss of *BRD7* function could not be assessed in humans. (ii) The detection of rare variants in patients with OZ was not performed, and the patients could carry different rare variants than those carried by patients with AZ. (iii) The selected tagSNPs captured 90% of the target alleles with r^2^ > 0.8 and MAF > 0.05, but they were not representative of all target alleles. (iv) Testicular *BRD7* levels of patients with severe spermatogenic impairment were not assessed due to ethical reasons. Our results require further validation in a larger cohort considering the limited number of participants in this study.

## Conclusions

In summary, this study is the first to investigate the association of the *BRD7* gene with spermatogenic failure and male infertility in humans. We failed to obtain any rare or common variant-based evidence for the significant influence of *BRD7* on spermatogenic efficiency and susceptibility in men, implying a limited contribution of the autosome-linked gene to spermatogenic failure and male infertility in humans.

## Supplementary Information

**Additional file 1: Figure S1.** Title: Sanger sequencing diagram of the rare variants. Legend: Sanger sequencing of rare variants predicted to potentially damage the function of bromodomain containing 7 (*BRD7*), including rs116422109, rs202057136, rs115302634 and rs188183810.

**Additional file 2: Table S1.** Primers for PCR and sequencing of *BRD7*. **Table S2.** Testing for Hardy-Weinberg equilibrium of the rare variants. **Table S3.** Testing for Hardy-Weinberg equilibrium of the common variants.

## Data Availability

The datasets used and analysed during the current study are available from the corresponding author on reasonable request.

## Abbreviations

BRD7: Bromodomain-containing protein 7
SNPs: Single nucleotide polymorphisms
AZF: Azoospermia factor
AZ: Azoospermia
OZ: Oligozoospermia
NZ: Normozoospermia
NOA: Nonobstructive azoospermia
SC: Sperm concentration
TSC: Total sperm count
FSH: Follicle-stimulating hormone
LH: Luteinizing hormone
T: Testosterone
PCR: polymerase chain reaction
gnomAD: Genome Aggregation Database
MAF: Minor allele frequency
HWE: Hardy-Weinberg equilibrium
LD: Linkage disequilibrium
SWI/SNF: Switch/sucrose nonfermenting
BRG1: Brahma-related gene 1
BRDT: Bromodomain testis-specific protein
