# Supplementary figures and images for: Is BRD7 associated with spermatogenesis impairment and male infertility in humans? A case-control study in a Han Chinese population
Source: Basic Clin Androl. 2021 Sep 2;31:19. doi: 10.1186/s12610-021-00139-3 (PMC8411525; doi:10.1186/s12610-021-00139-3)

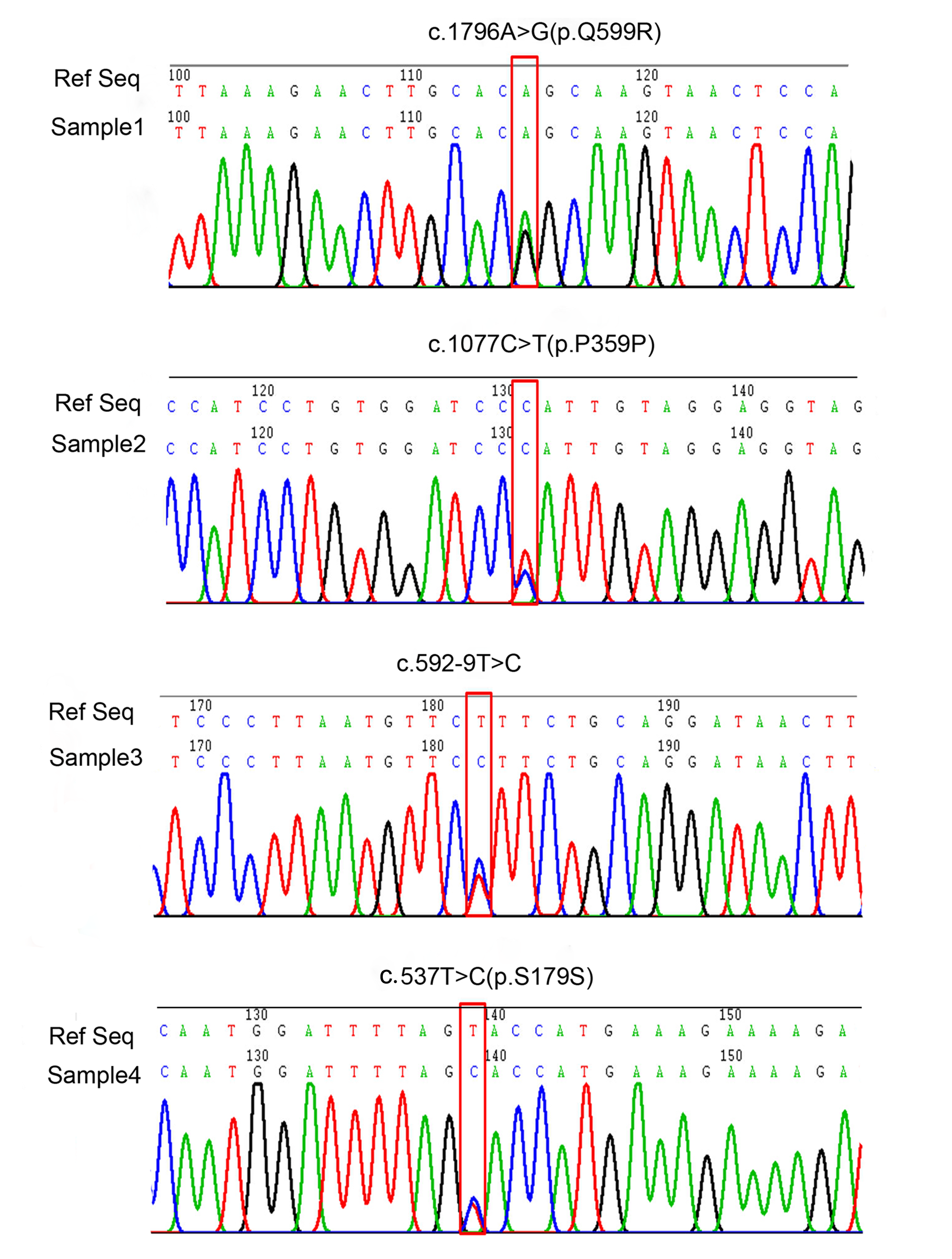

Supplement: Supplementary file 1 — Additional file 1: Figure S1. Title: Sanger sequencing diagram of the rare variants. Legend: Sanger sequencing of rare variants predicted to potentially damage the function of bromodomain containing 7 (BRD7), including rs116422109, rs202057136, rs115302634 and rs188183810. [file 12610_2021_139_MOESM1_ESM.tif]
