# Supplementary material for: Is BRD7 associated with spermatogenesis impairment and male infertility in humans? A case-control study in a Han Chinese population
Source: Basic Clin Androl. 2021 Sep 2;31:19. doi: 10.1186/s12610-021-00139-3 (PMC8411525; doi:10.1186/s12610-021-00139-3)
Supplement: Supplementary file 2 — Additional file 2: Table S1. Primers for PCR and sequencing of BRD7. Table S2. Testing for Hardy-Weinberg equilibrium of the rare variants. Table S3. Testing for Hardy-Weinberg equilibrium of the common variants. [file 12610_2021_139_MOESM2_ESM.docx]

**Table S1** Primers for PCR and sequencing of *BRD7*

| **Exons** |  | **Forward primers (5’-3’)** |  | **Reverse primers (5’-3’)** |  | **Amplicon size (bp)** |
| --- | --- | --- | --- | --- | --- | --- |
| Exon 1 |  | AGCTGCAGCACCACCTCC |  | CTCCCCACCAGAGACCCA |  | 579 |
| Exon 2 |  | CGGGGTCCGTCATTCAAC |  | AGGCGTTTGTTTTCCCCA |  | 455 |
| Exon 3-4 |  | CTGGCATAGAGACCCCCT |  | CGTATGTTTGGAGTTAGGCA |  | 791 |
| Exon 5 |  | CATCCTGAGGGTACAGCTAGT |  | TTTCATCTGGTTTTCCATCAC |  | 535 |
| Exon 6 |  | TTCATTCTGGGATCTACAGTTTA |  | GCTTCTGACAGGACTGACCA |  | 755 |
| Exon 7 |  | ATAGGGCAATAAAGTTGTAAAGC |  | GCTTTCTGAACTGTGTTTGGAC |  | 629 |
| Exon 8 |  | GTTAGGTGTGCCTTACTGTTTCT |  | ACATCCAACACGCCCACT |  | 553 |
| Exon 9 |  | CAATCCACAACACTCTGGTATC |  | GTTAGGCTAGCTGTTACCATGA |  | 340 |
| Exon 10 |  | AACAGGTCTGAGTTTTGGTCC |  | AATGTTGCAAAAATACCACCA |  | 435 |
| Exon 11 |  | GCAAACCAGCCAATGAAG |  | CAGATGCTACTATCAGGGAGACA |  | 435 |
| Exon 12 |  | GGAGCAGAAGATCCAGGGA |  | TTCCGTTTATTGTCCACCC |  | 537 |
| Exon 13 |  | CCCCAGCTCACAGAATCC |  | GCTAAAAACTTGTGGGGGA |  | 451 |
| Exon 14 |  | AAAAGGGTGTTCTGGAAATTCA |  | TTGGTTAGCCTTCTCCCTTC |  | 469 |
| Exon 15-16 |  | TAAATGAATGGGTATGGCGT |  | GGGTAAATACCAAATCCGAG |  | 763 |
| Exon 17 |  | CCTGTGTGCAGAAGAGCCA |  | GAAGCATTGGAAGGCACTAT |  | 484 |

**Table S2** Testing for Hardy-Weinberg equilibrium of the rare variants

| **Rs** |  | **Allele^a^** |  | **Hardy-Weinberg equilibrium testing** | |
| --- | --- | --- | --- | --- | --- |
|  |  |  |  | **Patients** | **Controls** |
| rs116422109 |  | T/C |  | 1.000 | 1.000 |
| rs202057136 |  | A/G |  | 1.000 | 1.000 |
| rs115302634 |  | A/G |  | 1.000 | 1.000 |
| rs188183810 |  | C/T |  | 1.000 | 1.000 |

^a^The minor alleles of the SNPs were underlined.

The Hardy-Weinberg equilibrium test was performed using PLINK 1.9 software.

**Table S3** Testing for Hardy-Weinberg equilibrium of the common variants

| **TagSNPs** |  | **Position_37** |  | **Location** |  | **Allele^a^** |  | **Hardy-Weinberg equilibrium testing** | |
| --- | --- | --- | --- | --- | --- | --- | --- | --- | --- |
|  |  |  |  |  |  |  |  | **Patients** | **Controls** |
| rs7196135 |  | 50353710 |  | intron16 |  | A/G |  | 0.331 | 0.489 |
| rs117164075 |  | 50363524 |  | intron8 |  | C/T |  | 0.787 | 0.496 |
| rs76946718 |  | 50363925 |  | intron8 |  | C/T |  | 0.711 | 0.847 |
| rs1062348 |  | 50368663 |  | synon_exon7 |  | G/A |  | 0.572 | 0.567 |
| rs79483509 |  | 50389470 |  | intron2 |  | T/C |  | 0.733 | 0.583 |
| rs62029995 |  | 50402240 |  | intron1 |  | C/G |  | 0.713 | 0.530 |
| rs11644238 |  | 50404393 |  | 5'-flanking |  | A/C |  | 0.737 | 1.000 |

^a^The minor alleles of the SNPs were underlined.

The Hardy-Weinberg equilibrium test was performed using PLINK 1.9 software. *SNP* Single nucleotide polymorphism
